# Food Insecurity and COVID-19: Disparities in Early Effects for US Adults

**DOI:** 10.3390/nu12061648

**Published:** 2020-06-02

**Authors:** Julia A. Wolfson, Cindy W. Leung

**Affiliations:** 1 Department of Health Management and Policy, University of Michigan School of Public Health, Ann Arbor, MI 48109, USA; 2 Department of Nutritional Sciences, University of Michigan School of Public Health, Ann Arbor, MI 48109, USA; cinleung@umich.edu

**Keywords:** covid-19, food insecurity, low-income adults, disparities, survey

## Abstract

The COVID-19 pandemic has dramatically increased food insecurity in the United States (US). The objective of this study was to understand the early effects of the COVID-19 pandemic among low-income adults in the US as social distancing measures began to be implemented. On 19–24 March 2020 we fielded a national, web-based survey (53% response rate) among adults with <250% of the federal poverty line in the US (*N* = 1478). Measures included household food security status and COVID-19-related basic needs challenges. Overall, 36% of low-income adults in the US were food secure, 20% had marginal food security, and 44% were food insecure. Less than one in five (18.8%) of adults with very low food security reported being able to comply with public health recommendations to purchase two weeks of food at a time. For every basic needs challenge, food-insecure adults were significantly more likely to report facing that challenge, with a clear gradient effect based on severity of food security. The short-term effects of the COVID-19 pandemic are magnifying existing disparities and disproportionately affecting low-income, food-insecure households that already struggle to meet basic needs. A robust, comprehensive policy response is needed to mitigate food insecurity as the pandemic progresses.

## 1. Introduction

Food insecurity, a condition defined by limited or uncertain access to sufficient, nutritious food for an active, healthy life, disproportionately affects low-income communities and communities of color [1]. Food is a core social determinant of health [2] and food insecurity is associated with numerous poor health outcomes in both the short and long term [3,4,5,6,7,8,9]. The unprecedented COVID-19 pandemic, and the associated social and economic response [10] (e.g., school closures, stay at home orders, business closures, and job losses) have the potential to dramatically increase food insecurity and its related health disparities among already at-risk populations. Early evidence suggests that food insecurity is indeed rapidly rising above pre-epidemic levels [11,12,13]. Household food insecurity has risen from 11% in 2018 to 38% in March 2020; in April 2020, 35% of households with a child aged 18 and under were food insecure [12,13]. Households already struggling with food insecurity may find their current situations exacerbated by COVID-19 with fewer resources to comply with social distancing recommendations. Food insecure individuals also may have less flexibility in their jobs to allow them to earn income while staying home, or may be at higher risk of losing their jobs completely, thereby decreasing (or eliminating) their incomes. These factors may put food insecure households both at higher risk of contracting COVID-19 and of greater food insecurity due to the economic effects of COVID-19 mitigation efforts.

In addition to the long-term health and economic effects of the COVID-19 pandemic, it is important to understand the immediate impact of social distancing measures to fight COVID-19 on vulnerable populations who already struggle to meet their basic needs. To do so, we fielded a national survey of low-income adults in the US on 19–24 March 2020 to understand the immediate effects of how COVID-19 was impacting low-income Americans and any disparities in its effects based on food security status.

## 2. Materials and Methods

We designed a web-based (Qualtrics) survey to measure the initial effects of COVID-19 on low-income adults in the United States (US) in mid-March 2020, just as some states were beginning to implement school closures and “stay at home” orders. The web-based survey was formatted to be accessible when access both via smart phones and on a personal computer or laptop. The survey was fielded using TurkPrime, an online crowdsourcing platform that is designed to be used for academic research [14]. TurkPrime allows researchers to use quotas to recruit a sample that matches their specific needs and has been used in numerous academic studies from a variety of disciplines published in the peer-reviewed literature [15,16,17,18,19]. In the present study, we used a census matched panel of US adults (matched on age, gender, and race/ethnicity to the overall population) and limited the sample to low-income adults with household incomes <250% of the federal poverty line (FPL). The FPL is calculated based on both household size and annual household income. For example, 100% of the FPL for a four-person household is $26,200, and 250% of the FPL for a four-person household is $655,000 per year. The annual income for a two-person household at 250% FPL is $43,100.

The survey was open to participants on 19–24 March 2020. We invited 2840 eligible panel members to participate and 1497 participants completed the survey (53% completion rate). Additional exclusions included participants who completed the survey in <4 min (*n* = 7), indicated they did not live in the US (*n* = 3), and were missing food insecurity data (*n* = 9) resulting in a final analytic sample size of 1478. Forty-four percent of participants took the survey on a personal computer or laptop and 56% took the survey on a smart phone or mobile device. This study was determined to be exempt by the Institutional Review Board at the University of Michigan.

### 2.1. Measures

*Food security:* Food security status over the past 30 days was measured using the 18-item US Household Food Security Module [20]. Questions are ordered by severity and include three levels of screening for adults, and an additional level of questions only for households with children. Affirmative responses to questions were summed to create a total food security score (out of 10 for adults and out of 18 for households with children). Food security categories (high, marginal, low, very low) were assigned according to US Department of Agriculture scoring guidelines [21]. The term food insecurity refers to the combined categories of low and very low food security.

*COVID-19-related basic needs challenges:* We inquired about challenges related to meeting basic needs people may have faced in the early weeks of the US COVID-19 epidemic and response. First, we asked about participants’ ability to comply with recommendations to purchase two weeks of food (which was recommended by public health efforts to limit grocery shopping trip and facilitate social distancing). We also asked participants whether they had encountered any of the following challenges due to the coronavirus: the ability to feed their family, availability of household items such as toilet paper, access to healthcare, access to medications, the ability to pay bills, ability to rent or pay mortgages, whether they had been unable to work due to lack of childcare, and whether they had been unable to work due to illness.

*COVID-19-related workplace reactions:* At the time of data collection, some, but not all, states had begun issuing stay at home orders and mandatory business closures. Even in states without stay at home mandates, some businesses were making adjustments to operations due to COVID-19. We asked working adults (i.e., those with full- or part-time work outside the home) what their employer was doing to adjust to the pandemic. Specifically, we asked “Workplaces in the US are adjusting to the coronavirus situation in different ways. What is your workplace doing to adjust?” Participants were given the following response options: nothing, my workplace is proceeding as normal; all employees are encouraged to work at home; all employees must work at home; essential employees must come in to work but others can work from home; hours are being reduced for hourly employees; my place of employment has temporarily closed due to the coronavirus; my place of employment has closed and I have been laid off; work is busier and employees need to work longer hours; other.

*Expected impact of COVID-19 on employment and income:* We asked working adults what they expected would happen at their job if they or someone in their family became ill with COVID-19. Response options focused on whether they would be able to stay home, whether they had vacation or sick days they could use, and what they expected would happen if they missed work due to illness.

### 2.2. Analysis

All analyses were conducted in 2020 with Stata, Version 15 (StataCorp LP, College Station TX, USA). First, we describe the socio-demographic characteristics of the study sample overall and by food security status using cross tabulations and chi-squared tests of significant differences. Next, we examine differences in COVID-19-related basic needs and workplace challenges (among participants working full or part time), by food security status using cross tabulations. Missing data was treated using listwise deletion. Significant differences by food security status were assessed using chi-squared tests. All tests were two tailed and significance was considered at *p* < 0.05.

## 3. Results

The characteristics of the sample of low-income adults are presented in Table 1. Overall, 36% of this sample was food secure, 20% had marginal food security, and 44% were food insecure (17% low food security; 27% very low food security). Individuals with low or very low food security were more likely to be non-Hispanic Black or Hispanic, to have children in the home, and have less than a college education. Individuals with very low food insecurity were also more likely to rent their homes, not have health insurance or have Medicaid, and were more likely to be receiving SNAP benefits. The distribution of the sample by state of residence is shown in Appendix A.

**Figure 1:**
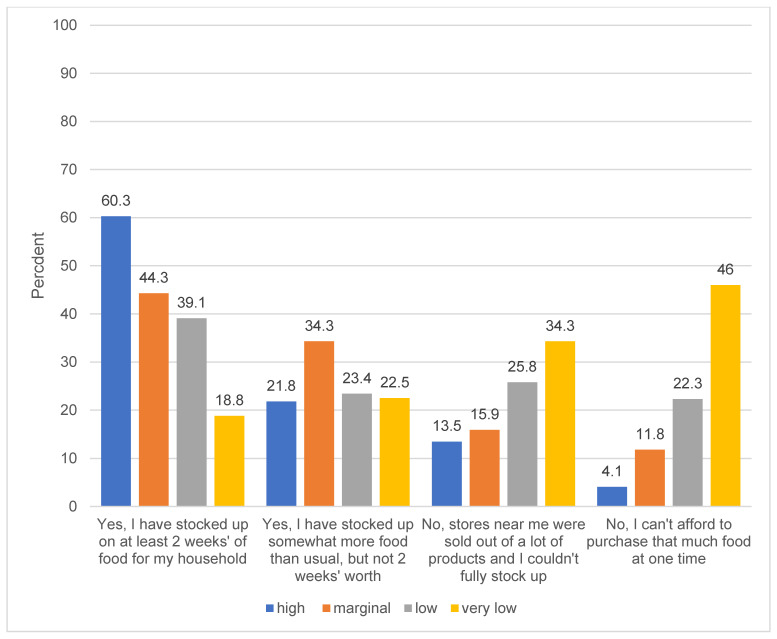
Ability to comply with recommendation to stock up on two weeks of food among low-income US adults, by food security status (*n* = 1478). Question text: “Experts have recommended stocking up on two weeks of food for your household to prepare for the coronavirus. Have you been able to do this? [Please check all that apply. One respondent was missing data for this question and was excluded from analysis. Differences within each response option by food security status are significant at *p* < 0.001 based on chi-squared tests.

Figure 1 shows the ability of low-income US adults to comply with public health recommendations to stock up on two weeks of food to avoid excess grocery store trips and facilitate social distancing. Nearly 2/3 (60%) of food-secure, low-income adults reported being able to comply with that recommendation, compared to less than one in five (18.8%) of low-income adults with very low food security. Adults with very low food security were more likely to report their local stores were sold out of products, and not being able to afford to purchase an extra two weeks of food at one time.

**Figure 2:**
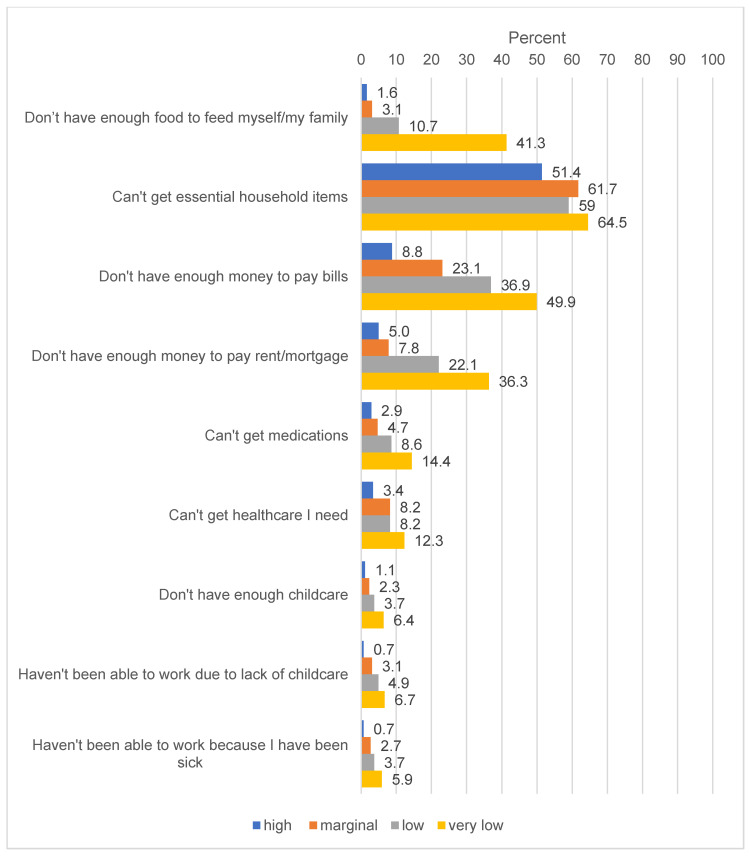
Challenges encountered by low-income US adults as a result of COVID-19, as of 19–24 March, by food security status (*n* = 1478). Question text: “Have you experienced any of the following challenges due to the coronavirus (COVID-19) so far?” [Please check all that apply] Percentages reflect the percent of respondents in each food security category that said they encountered that problem. Ten percent of respondents (*n* = 161) did not indicate any of the response options were challenges for them and are counted as ‘missing’.

Potential basic needs challenges related to COVID-19 are displayed in Figure 2. For every challenge asked about, food-insecure adults were significantly more likely to report dealing with that challenge, with a clear gradient effect based on severity of food security status. Strikingly, 41.3% of adults with very low food security reported not having enough food to feed themselves or their family compared to 10.7% of adults with low food security, 3.1% of adults with marginal food security and 1.6% of adults with high food security. Half (49.9%) of adults with very low food security did not have enough money to pay their bills compared to 36.9% of those with low food security, 23.1% of those with marginal food security and 8.8% of food secure adults.

Food-secure, low-income adults working full or part time (44.3% of the overall sample) were more likely than their food-insecure counterparts to work in jobs that were either proceeding as normal, were busier than usual, or had closed and laid off employees. In contrast, working adults with food insecurity were more likely to have their hours reduced (Table 2). When asked what they thought would happen if they or someone in their family got sick with COVID-19, working adults with very low food security were less likely than their food-secure counterparts to have sick days or vacation days they could use, and were more likely to say they would lose their job if they missed too many days of work (52% very low food security vs. 18% high food security, *p* < 0.001).

## 4. Discussion

This study presents results from a national survey of low-income adults in the US in the days immediately following the first major policy steps to enforce COVID-19-related social distancing measures on a wide scale in the US. Though large-scale school and business closures were only beginning to be implemented [22], we find that the effects of the COVID-19 pandemic were already impacting low-income adults, with disproportionately negative effects for low-income adults experiencing food insecurity. This initial evidence from a time period before even greater economic effects and job losses took place demonstrate that the COVID-19 pandemic threatens to greatly exacerbate existing health disparities related to food security status. Indeed, evidence from later surveys show that food insecurity in the US has dramatically increased well beyond levels seen during the Great Recession [12,13].

Results from this study illuminate the extent to which, very early in the COVID-19 trajectory in the US, individuals with food insecurity were disproportionately vulnerable to the severe economic and health consequences of the crisis. Our findings show that as early as mid-March 2020, food-insecure adults currently working outside the home were at greater risk of losing their income or their jobs if they got sick from COVID-19. Regardless of whether they get sick or their employment status, food-insecure individuals were also more likely to report expecting that they will lose income during the pandemic. For already low-income households, loss of income puts them at high risk of severe food insecurity and an inability to meet other basic needs, both of which can lead to future physical and mental health problems [23,24,25,26]. Compared to low-income, food-secure adults, food-insecure adults were more likely to report that they had already been laid off, and that their income would go down substantially. Fifty-four percent of food secure adults reported they expected their income would remain the same compared to 23% of adults with very low food security (results not shown, but available upon request). Subsequent massive job losses [27] and more extensive social distancing measures [10] after data collection ended have likely exacerbated the trends we document in our results.

Across the lifespan, food insecurity is associated with a range of negative health outcomes over the short and long term, including poor mental health outcomes such as depression, stress, and anxiety [4,9,28], poor diet quality [7,29], high rates of chronic diseases such as diabetes and obesity [6,30,31], and lower overall health status [3,5,32]. Food insecurity is also associated with higher healthcare expenditures, in part due to the higher burden of chronic health conditions among food-insecure patients and the known tradeoffs between food and medicine [24,26]. As the COVID-19 pandemic and the associated economic fallout progress, it will be critical for policymakers, health systems, and the public health community to proactively and comprehensively address access to food and other basic needs, particularly for populations at risk of, and already experiencing, food insecurity. Failure to do so will have long-term implications for population health, health disparities, and the health care system as a whole.

Food-insecure adults are more likely to be people of color, with lower social standing, who have less flexible and secure jobs, and are more vulnerable to chronic stress and other basic needs insecurities [1]. In 2018, 11.1% of adults in the US were food insecure; among low-income adults (<185% FPL), 29.1% were food insecure [1]. We find that, as of mid-March, 2020, 44% of adults with an income <250% of the FPL were food insecure in the past 30 days, and these individuals were more likely to be non-Hispanic Black or Hispanic. This disparity in food security status based on race/ethnicity is an additional way in which COVID-19 is disproportionately impacting communities of color in the US. Since mid-March, adult and child food insecurity rates in the US have dramatically risen [12,13]. In our study, adults currently experiencing food insecurity were not able to buy food in bulk quantities and therefore are at greater risk of exposure to the virus (due to the need for more frequent food shopping trips) as well as being at greater risk of an acute hunger crisis (due to lack of financial resources to purchase sufficient food). In addition, as individuals already at risk for food insecurity are more vulnerable to losing their jobs, rates of food insecurity will climb higher as the pandemic progresses.

Direct income support, expanded unemployment benefits, and additional support for federal food assistance programs included in the CARES act (passed on 27 March 2020) and the Families First Coronavirus Response Act (passed on 18 March 2020) are important first steps to supporting low-income families in the US [33,34]. However, longer-term support for individuals, as well as institutions and organizations that provide food, is needed. Some communities are already experiencing unprecedented demand in the emergency food system [35], and the federal government, states, and cities are scrambling to ensure that families with children who depend on free or reduced price meals at school do not go hungry [36]. Given the scale of the pandemic and the likely duration of social distancing measures and the associated economic impacts, more support is urgently needed to mitigate the toll of COVID-19 on the most vulnerable members of society. In particular, in addition to direct economic support to individuals, financial support for the emergency food system, greater flexibility for school systems to provide food to families, and long-term, expanded food assistance support via the Supplemental Nutrition Assistance Program (SNAP) are all urgently needed. Expanded SNAP benefits were critical for providing needed support for low-income families during the Great Recession, and were effective at reducing food insecurity [37]. Congress and the Trump administration should urgently increase SNAP benefits and expand eligibility for the program to help low-income families afford food during this extraordinary time.

### Limitations

Results from this study should be considered in light of some limitations. First, the web-based survey panel does not use probability-based sampling and is not nationally representative. However, the TurkPrime panel is national in scope, and uses census-matched quotas to achieve a sample that closely aligns with the demographics of the population in the US which mitigates some of this concern. However, because we limited our sample to households <250% of the FPL (based on income and household size), and because the survey was only available in English, the demographics of our sample may be more similar to the US population overall and undercount some key demographic groups, particularly non-Hispanic Blacks and Hispanics, non-English speakers, and immigrants. Relatedly, this data was collected via a web-based survey which by definition required participants to have internet access via a computer or a smart phone. This method of data collection could also have undercounted some groups (e.g., those with very low income, without high school degrees, and those living in rural areas without broadband internet access) [38], likely those especially vulnerable to food insecurity. It is important to note, however, that any bias introduced from these factors would have biased results into the direction of undercounting, rather than overcounting, food insecurity and the other outcomes we document here. Second, respondents could choose whether or not to participate in the survey which may introduce some selection bias. Third, all measures in the study are self-reported and may be subject to a social-desirability bias. However, the fact that the survey was fielded online and was completely anonymous may mitigate this concern. Fourth, this survey is cross-sectional and we cannot make any statements about causal relationship between the coronavirus and our measure of food insecurity in the past 30 days. Finally, data were collected very quickly after social distancing measures and business and school closures began to be implemented in some (but not all) states. This is a strength as we were able to capture the immediate, real-time impacts on low-income adults. However, some measures also focused on anticipated effects. It is possible that the respondents did not accurately assess the likely effect of the coronavirus pandemic on their employment and income. However, initial evidence in the weeks after our data were collected show clearly that unemployment and rates of food insecurity have skyrocketed. The longer-term effects on low-income adults in the US, and associated disparities based on food insecurity, may be better or worse than those expected by participants in this survey. It will be imperative for future research to examine the long-term effects of the coronavirus pandemic and associated social distancing measures on food insecurity and associated health outcomes, particularly among vulnerable communities that were already struggling at the start of the pandemic.

The strengths of our study include the fact that we were able to collect these data so quickly after a national emergency was declared and states began implementing policies to slow the spread of COVID-19. Our large national sample of low-income adults is another key strength as is our use of the gold standard 18-question USDA food security screener module.

## 5. Conclusions

The social and economic upheaval caused by the COVID-19 pandemic is magnifying existing disparities and disproportionately affecting low-income, food-insecure households that already struggle to meet basic needs. The early effects documented in the present study are likely to continue to worsen as the pandemic continues unless extensive policy and economic supports are swiftly implemented.

## Figures and Tables

**Table 1:** Demographic characteristics of the study sample overall and by food security status (*n* = 1478).

	Overall	Food Security Status				*p*-Value
		High	Marginal	Low	Very Low	
	*n* (%) ^a^	*n* (%) ^b^	*n* (%) ^b^	*n* (%) ^b^	*n* (%) ^b^	
Total	1478 (100)	532 (36)	290 (20)	256 (17)	400 (27)	
Age						
18–39	635 (43)	168 (26)	116 (18)	140 (22)	211 (33)	<0.001
40–59	429 (29)	152 (35)	88 (21)	62 (14)	127 (30)	
≥60	414 (28)	212 (51)	86 (21)	54 (13)	62 (15)	
Sex						
Male	733 (50)	285 (39)	135 (18)	128 (17)	185 (25)	0.100
Female	745 (50)	247 (33)	155 (21)	128 (17)	215 (29)	
Race/ethnicity						
NH White	990 (67)	384 (39)	185 (19)	160 (16)	261 (26)	0.026
NH Black	161 (11)	47 (29)	36 (22)	36 (22)	42 (26)	
Hispanic	186 (13)	55 (30)	35 (19)	39 (21)	57 (31)	
Asian	73 (5)	24 (33)	23 (32)	11 (15)	15 (21)	
Other	68 (5)	22 (32)	11 (16)	10 (15)	25 (37)	
Household Size						
1–3 people	1113 (75)	416 (37)	219 (20)	177 (16)	301 (27)	0.054
≥4 people	365 (25)	116 (32)	71 (19)	79 (22)	99 (27)	
Marital Status						
Single, never married	564 (38)	199 (35)	108 (19)	118 (21)	139 (35)	<0.001
Married	448 (30)	180 (40)	91 (20)	68 (15)	109 (24)	
Separated, divorced, widowed	311 (21)	124 (40)	58 (19)	43 (14)	86 (28)	
Living with a partner	150 (10)	27 (18)	32 (21)	26 (17)	65 (43)	
Children < 18 years in home						
Yes	445 (30)	120 (27)	85 (19)	92 (21)	148 (33)	<0.001
No	1033 (70)	412 (40)	205 (20)	164 (16)	252 (24)	
Income						
<$35,000/year	894 (60)	297 (33)	175 (20)	165 (18)	257 (29)	0.015
$35,000 ≤ $59,000/year	418 (28)	162 (39)	75 (18)	69 (17)	112 (27)	
≥$59,000/year	166 (11)	73 (44)	40 (24)	22 (13)	31 (19)	
Education						
High school/GED	439 (30)	122 (28)	83 (19)	91 (21)	143 (33)	<0.001
Some college	524 (35)	197 (38)	104 (29)	75 (14)	148 (28)	
College/grad degree	515 (35)	213 (41)	103 (20)	90 (17)	109 (21)	
Employment status						
Full time job (hourly or salary)	408 (29)	139 (34)	68 (17)	81 (20)	120 (29)	0.002
Part time job (hourly or salary)	239 (17)	83 (35)	51 (21)	41 (17)	64 (27)	
Not working, looking for work	197 (14)	58 (29)	38 (19)	38 (19)	63 (32)	
Not working, not looking for work	415 (30)	186 (45)	86 (21)	55 (13)	88 (21)	
Home-maker	141 (10)	46 (33)	27 (19)	21 (15)	47 (33)	
Student						
Yes	95 (6)	29 (31)	26 (27)	20 (21)	20 (21)	0.106
No	1383 (94)	503 (36)	264 (19)	236 (17)	380 (27)	
Home ownership						
Rent	744 (50)	201 (27)	144 (19)	154 (21)	245 (33)	<0.001
Own	538 (43)	287 (45)	128 (20)	89 (14)	134 (21)	
Other	96 (7)	44 (46)	18 (19)	13 (14)	21 (22)	
Health insurance						
None	231 (16)	68 (29)	40 (17)	35 (15)	88 (38)	<0.001
Yes, through work	260 (18)	97 (37)	45 (17)	57 (22)	61 (23)	
Yes, Medicare	437 (30)	189 (43)	83 (19)	73 (17)	92 (21)	
Yes, Medicaid	338 (23)	91 (27)	73 (22)	55 (16)	119 (35)	
Yes, other	212 (14)	87 (41)	49 (23)	35 (17)	40 (19)	
Political party affiliation						
Republican	396 (27)	174 (44)	76 (19)	50 (13)	96 (24)	0.004
Democrat	594 (40)	190 (32)	124 (21)	115 (19)	165 (28)	
Independent	488 (33)	168 (34)	90 (18)	91 (19)	139 (28)	
SNAP benefits						
No	1065 (72)	452 (42)	207 (19)	182 (17)	224 (21)	<0.001
Yes	413 (28)	80 (19)	83 (20)	74 (18)	176 (43)	
Region of residence						
Northeast	273 (18)	90 (33)	57 (21)	59 (22)	67 (25)	0.406
Midwest	332 (22)	127 (38)	69 (21)	47 (14)	89 (27)	
South	542 (37)	196 (36)	95 (18)	97 (18)	154 (28)	
West	331 (22)	119 (36)	69 (21)	53 (16)	90 (27)	

^a^ Column percentage; ^b^ Row percentage.

**Table 2:** COVID-19 effects on workplaces among low-income adults working full or part time in the US overall and by food security status as of 19–24 March 2020 (*n* = 655).

	Overall (*n* = 655)	Food Security Status				*p*-Value
		High *n* = 225 (34%)	Marginal *n* = 120 (18%)	Low *n* = 124 (19%)	Very Low *n* = 186 (28%)	
	*n* (%) ^a^	*n* (%) ^b^	*n* (%) ^b^	*n* (%) ^b^	*n* (%)^b^	
What is your workplace doing to adjust to COVID-19? ^c^						
Nothing, proceeding as normal	152 (23)	64 (42)	27 (18)	25 (16)	36 (24)	0.004
Employees encouraged to work at home	69 (11)	15 (22)	17 (25)	17 (25)	20 (29)	
Employees must work at home	69 (11)	17 (25)	14 (20)	16 (23)	22 (32)	
Essential employees must come in, others work from home	58 (9)	29 (50)	5 (9)	14 (24)	10 (32)	
Hours are reduced	79 (12)	20 (25)	11 (14)	17 (22)	31 (39)	
Temporarily closed	131 (20)	44 (34)	27 (21)	21 (16)	39 (30)	
Closed and I have been laid off	25 (4)	11 (44)	6 (24)	3 (12)	5 (20)	
Busier, employees working extra hours	47 (7)	21 (45)	9 (19)	8 (17)	9 (19)	
If you or someone in your family becomes ill with COVID-19, what do you expect will happen regarding your job? ^c^ (check all that apply)						
I will be able to stay home without using sick or vacation days	162 (26)	71 (44)	31 (19)	31 (19)	29 (18)	0.003
I will be able to use sick days to stay home without losing income	123 (19)	55 (45)	22 (18)	23 (19)	23 (19)	0.022
I will be able to use vacation days to stay home without losing income	74 (12)	27 (36)	11 (15)	17 (23)	19 (26)	0.573
I do not have sick days so if I am not able to work I will lose income	260 (41)	72 (28)	49 (19)	45 (17)	94 (36)	0.002
I will have to go into work even if I am sick	33 (5)	6 (18)	6 (18)	8 (24)	13 (39)	0.180
If I miss too many days of work I could lose my job	61 (10)	11 (18)	9 (15)	9 (15)	32 (52)	<0.001

^a^ Column percentage; ^b^ Row percentage; ^c^ Asked among respondents who are working full or part time.

## Appendix A

**Table A1:** Distribution of the Sample by State.

State	*N*	Percent
Alabama	22	1.49
Alaska	3	0.2
Arizona	35	2.37
Arkansas	16	1.08
California	150	10.15
Colorado	19	1.29
Connecticut	10	0.68
Delaware	7	0.47
District of Columbia	2	0.14
Florida	102	6.9
Georgia	50	3.38
Hawaii	7	0.47
Idaho	8	0.54
Illinois	62	4.19
Indiana	28	1.89
Iowa	11	0.74
Kansas	19	1.29
Kentucky	27	1.83
Louisiana	17	1.15
Maine	10	0.68
Maryland	20	1.35
Massachusetts	26	1.76
Michigan	58	3.92
Minnesota	21	1.42
Mississippi	14	0.95
Missouri	30	2.03
Montana	4	0.27
Nebraska	5	0.34
Nevada	26	1.76
New Hampshire	6	0.41
New Jersey	37	2.5
New Mexico	13	0.88
New York	100	6.77
North Carolina	52	3.52
North Dakota	0	0
Ohio	68	4.6
Oklahoma	25	1.69
Oregon	17	1.15
Pennsylvania	72	4.87
Puerto Rico	0	0
Rhode Island	3	0.2
South Carolina	30	2.03
South Dakota	3	0.2
Tennessee	31	2.1
Texas	90	6.09
Utah	21	1.42
Vermont	3	0.2
Virginia	29	1.96
Washington	28	1.89
West Virginia	13	0.88
Wisconsin	28	1.89
Wyoming	0	0
**Total**	**1478**	**100**
